# Attentional and Contextual Priors in Sound Perception

**DOI:** 10.1371/journal.pone.0149635

**Published:** 2016-02-16

**Authors:** Michael Wolmetz, Mounya Elhilali

**Affiliations:** 1 Applied Neuroscience Section, Johns Hopkins University Applied Physics Laboratory, 11100 Johns Hopkins Rd, Laurel, MD 20723, United States of America; 2 Department of Electrical and Computer Engineering, Johns Hopkins University, 3400 N Charles Street, Barton Hall, Rm 105, Baltimore, MD 21218, United States of America; UNLV, UNITED STATES

## Abstract

Behavioral and neural studies of selective attention have consistently demonstrated that explicit attentional cues to particular perceptual features profoundly alter perception and performance. The statistics of the sensory environment can also provide cues about what perceptual features to expect, but the extent to which these more implicit contextual cues impact perception and performance, as well as their relationship to explicit attentional cues, is not well understood. In this study, the explicit cues, or attentional prior probabilities, and the implicit cues, or contextual prior probabilities, associated with different acoustic frequencies in a detection task were simultaneously manipulated. Both attentional and contextual priors had similarly large but independent impacts on sound detectability, with evidence that listeners tracked and used contextual priors for a variety of sound classes (pure tones, harmonic complexes, and vowels). Further analyses showed that listeners updated their contextual priors rapidly and optimally, given the changing acoustic frequency statistics inherent in the paradigm. A Bayesian Observer model accounted for both attentional and contextual adaptations found with listeners. These results bolster the interpretation of perception as Bayesian inference, and suggest that some effects attributed to selective attention may be a special case of contextual prior integration along a feature axis.

## Introduction

Perception of our surrounds is not merely a reflection of sensory information instantly gathered from the environment. It is an active process that is very sensitive to numerous factors, including attentional state and expectations. For example, explicit attentional cues can rapidly enhance perception of expected events (e.g. a specific color [1] or frequency region [2]) and remarkably, leave many observers virtually blind or deaf to unexpected visual or acoustic events [3,4]. These examples all involve manipulating the observers’ expectations *explicitly*: attend to a specific color, location in space, tone frequency, a female voice, etc.

Expectations can also arise from *implicit* cues learned from our sensory surrounds over multiple timescales, ranging from very recent experiences to the course of a lifetime. Some expectations, like those related to the structure of natural images [5] or the phonetic features [6,7] of one’s language, are more or less entrenched by adulthood and continuously guide everyday perception of object and words. Other expectations are formed over the very recent past. In vision, strong biases to particular directions of motion can be induced over the course of a few minutes [8]. In audition, similar biases have been induced over similar time scales with respect to perception of tone frequency and melody [9,10]. Despite these limited reports, the study of sensory expectations remains in its infancy. The implicit nature of these expectations as well as the nested time-constants over which they arise make them difficult to investigate. Their behavioral consequences are also often linked to and sometimes confounded with those of more explicit factors such as selective attention; in that both mechanisms often result in heightened perception of attended or ‘expected’ stimuli [11,12].

Despite these potential confounds, the perceptual changes induced by expectations or beliefs have been successfully formalized and quantified using Bayesian models [13–17]. Bayesian inference is particularly appropriate for capturing the diversity of phenomena described above, both in terms of explicit and implicit cues, as well as the complex nature of dynamics over which they operate. To begin with, Bayesian perceptual estimates combine sensory evidence for a perceptual feature with the expectation or prior probability of that feature. Given some sensory uncertainty or noise, incorporating priors improves perception when the priors accurately reflect the statistics of the current environment, and may corrupt perception when they do not. Furthermore, Bayesian models are agnostic to the sources of priors (i.e. attentional cues or sensory contexts) and the speed of their collection (over the lifespan, an hour, or a single trial). This interpretation of perception as Bayesian inference has a powerful implication: that priors are collected and integrated across many perceptual features, sources, and time scales to the overall benefit of the observer. Of course, exploring this implication is a collective effort across many domains.

The current study uses a standard auditory detection task to test several predictions of a Bayesian model of auditory scene analysis, with particular attention paid to the interplay between implicit contextual cues and explicit attentional cues in biasing perception. The first and most basic prediction of a Bayesian model of auditory scene analysis is that contextual priors present in the recent statistical history will be tracked (across a variety of acoustic stimuli) and will bias perception. We tested this prediction by way of a well-studied effect of auditory selective attention, the *attentional band*.

**Fig 1:**
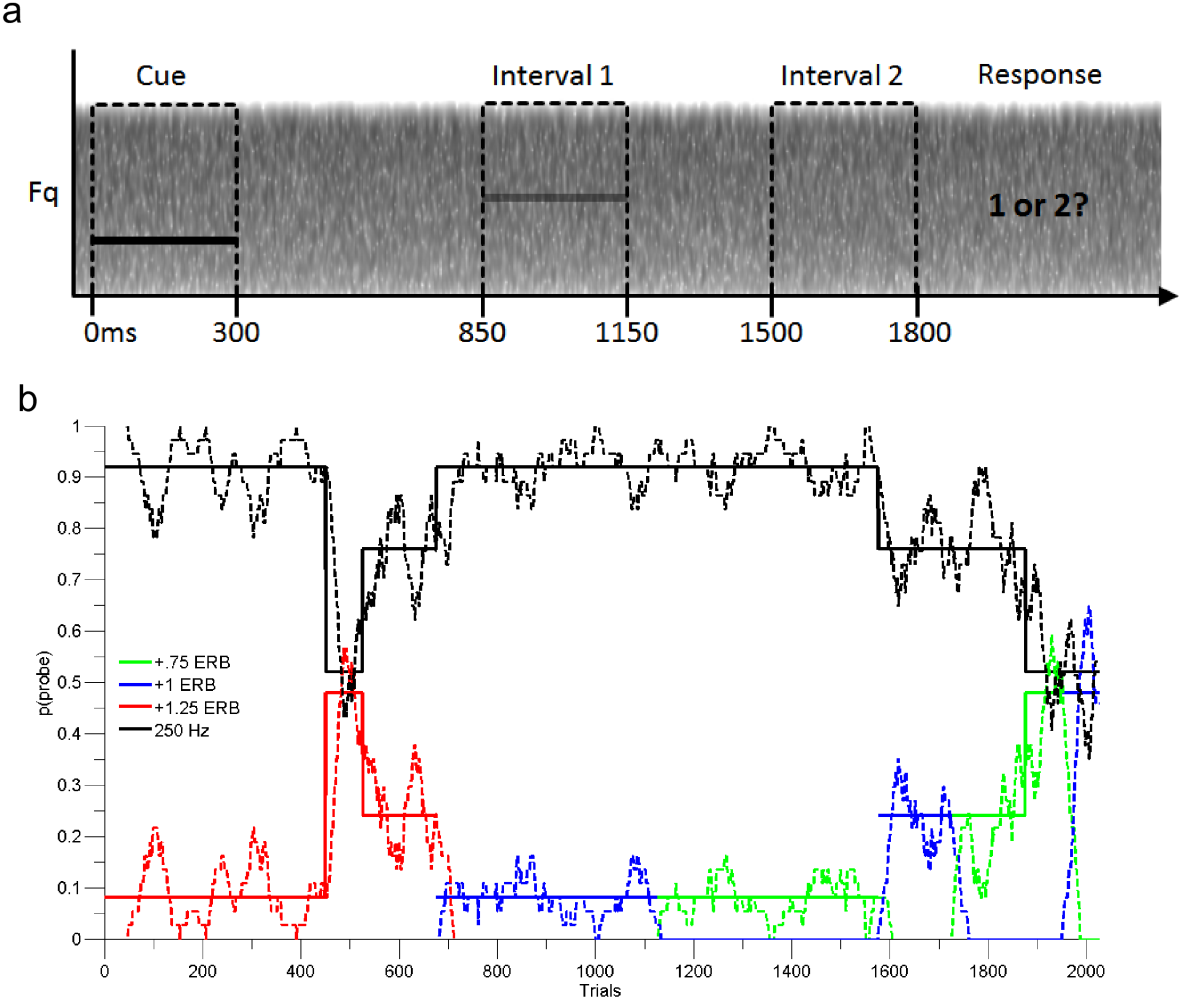
Summary of behavioral paradigm. (a) Trial schematic for cued two-interval forced-choice probe-signal method. Participants reported the interval in which they perceived a sound over a continuous noise masker. Cue sounds were presented at clearly audible levels, detection sounds were presented near threshold. (b) Randomized stimulus sequence for one subject. Solid lines show the discrete levels of probability used to generate stimulus sequences plotted over time, and dotted lines show the resulting continuous levels of stimulus probability calculated from recent history (40 trials).

When listeners are asked to detect the presence of a tone (any tone) in noise, a high explicit attentional prior placed on a particular tone frequency via cueing causes an attentional band of perceptual sensitivity around that frequency. This means that tone detection rates fall off as a function of frequency distance from the expected tone(s) [18–23]. Similar to previous studies of the attentional band, the current study used a version of the probe-signal procedure [24] in which listeners are asked to detect simple tones at near-threshold levels in a background of continuous white noise (Fig 1a). Here, a 250 Hz tone was present during the majority of two-interval forced choice detection trials. Infrequent probe tones differing from the cued frequency (at 291, 305, 319, 350, and 487 Hz) were presented in a minority of trials, and the prior probability associated with each probe frequency was varied throughout the experiment (Fig 1b). If listeners are implicitly tracking the contextual priors associated with tone frequency, as a Bayesian model of auditory scene analysis would predict, tone detection rates should fall off as a function of decreased prior probability. This prediction was tested for simple tones, as well as harmonic stacks and English vowels.

A second prediction of a Bayesian model of auditory scene analysis is that listeners collect and use contextual priors optimally. In a dynamic environment where context changes and the prior probabilities associated with different tone frequencies vary over time, a Bayesian model predicts that past evidence will be weighted in such a way as to provide the best estimates of the future. This prediction was tested by analyzing tone detection rates as a function of prior probability as computed over different windows sizes and decay parameters to determine how contextual priors are integrated over time.

The final prediction of a Bayesian model of auditory scene analysis tested here is that listeners use explicit attentional cues and implicit contextual cues simultaneously and independently. Both attentional and contextual cues have been shown to bias perception, but (as will be demonstrated) Bayesian observer models predict contextual priors have the same effect on detection sensitivity whether there is an effective attentional prior, an ineffective attentional prior, or no attentional prior at all. This prediction was tested by explicitly cuing listeners to expect that a 250Hz frequency would regularly (but not always) occur, forming a simple and explicit attentional prior during the prior probability manipulation.

## Materials and Methods

### Probe-signal procedure

The procedure closely followed the probe-signal method used by Dai and colleagues [20]. Each trial consisted of a cued two-interval forced choice detection in which listeners were instructed to report whether a sound occurred (over a continuous noise masker) in the first or second of two observation intervals. Listeners were additionally instructed that the cue sound marked the beginning of the trial. In all versions except for the uninformative cue version of the task, listeners were also told that the cue provided a hint about the sound *likely* to appear in that trial, with corresponding examples and practice. The specific instructions given were: “The cue sound will often be the same sound that appears in the trial. Occasionally the cue sound will not be the same sound that appears in the trial”. The cue, two intervals, and an opportunity for response were indicated by text appearing sequentially on a monitor at the appropriate times.

Cue sounds were presented at clearly audible levels, and the sound presented for detection was attenuated at its threshold level in each trial and with equal probability in the two intervals (550 ms or 1200 ms after cue offset). One of four different detection sounds were presented in each trial: a sound matching the cue in pitch, or a probe sound differing from the cue by one of three distances along the frequency axis. For all three stimulus classes tested (simple tone detection, harmonic stack detection, and vowel detection), the cued pitch was 250 Hz and probe pitches were .75 Equal Rectangular Bandwidths (ERB, as calculated from [25]), 1 ERB, and 1.25 ERBs from the cued pitch, corresponding to 291 Hz, 305 Hz, and 319 Hz respectively. For the simple tones only, two additional data sets were collected: (1) an *uninformative cue* version in which the detection tones were as described but an uninformative cue was presented at 777 Hz and related cue instructions were omitted, (2) the standard informative cue version for additional probe distances of 1.75 ERB (350 Hz) and 3.75 ERB (487 Hz) to more fully fit the attentional band.

In addition to variations along frequency or perceived pitch, the prior probabilities of each sound also varied. Each probe pitch was presented with a probability of .08, .24, and .48, and the cued pitch (or simply the *probable pitch* in the case of the uninformative cue) was presented in the remaining proportion of trials (.92, .76, and .52) in a given condition. Each condition was made up of blocks of 75 trials each, and prior probabilities were calculated over this length. In this way, each condition contained two detection sounds: one with a pitch of 250 Hz presented with some probability *p* and another with a different pitch presented with a probability of *1 –p* (Fig 1b). In summary, experiment had nine conditions: three levels of probe distance crossed with three levels of probe probability. For each of those nine conditions, there were 36 total probe trials. For example, for the conditions where probe probability was low (.08), there were 414 target trials associated with this condition and 36 probe trials, for a total of 450 trials presented over six consecutive blocks. Participants were given the option to rest between blocks, and heard a total of 2025 total trials each.

### Threshold measurements

Thresholds of approximately 90% accuracy in continuous noise for all acoustic stimuli were estimated for each listener using an adaptive staircase procedure [26] for the 250 Hz stimulus followed by block thresholding procedures for all stimuli. The adaptive staircase procedure proceeded up and down in intensity with decreasing step sizes for 15 reversals; the adaptive threshold was calculated as the mean intensity of trials after the first three reversals. This threshold was then used as a starting threshold for a block thresholding procedure, in which the signal level was adjusted after each block of 20 trials until a block accuracy of 85% to 95% was reached. Thresholding was effective in producing comparable accuracy across stimuli within each class: Simple tones [mean threshold accuracy, standard deviation]: 250 Hz [90.6%, 4.2%], 291 Hz [90.7%, 4.1%], 305 Hz [89.5%, 3.8%], 319 Hz [91.3%, 3.8%], 350 Hz [90.5%, 4.2%], 487 Hz [89.4%, 3.9%]. Harmonic stacks: 250 Hz [92.3%, 4.2%], 291 Hz [92.3%, 4.2%], 305 Hz [89.7%, 3.5%], 319 Hz [92.0%, 4.1%]. Vowels: 250 Hz [91.5%, 4.1%], 291 Hz [93.0%, 3.5%], 305 Hz [92.0%, 4.2%], 319 Hz [92.5%, 3.5%]. In no cases were block threshold accuracies significantly different (p < .05) for different stimuli of the same class.

### Post-experiment interview

Listeners were interviewed at the conclusion of probe-signal testing in an effort to assess whether they remembered being aware of the distance or probability manipulation, and whether they deliberately adopted any subsequent strategies. Listeners were asked: (1) “Did you notice anything in particular about how often the different sounds were played? If so, what did you notice?” (2) “Did you use any particular strategy to detect the sound? If so, can you describe your strategy?” No listener responded in any way consistent with conscious awareness of the probability manipulation or any deliberate detection strategy related to tone probability.

### Stimuli and equipment

All stimuli had a duration of 300ms, a sampling rate of 16 kHz, began and ended at zero crossings, and were presented with 25ms cosine rise and fall times. Simple tones (single sinusoids) and harmonic spectra (10 odd harmonics, h = 1:10, stacked with decreasing relative amplitudes of 1/h) were generated in MATLAB and vowels (/a:/ as in father) were generated with the Klatt Synthesizer. While the same formant parameters were used to generate all vowels (F1 = 936 Hz, F2 = 1551 Hz, F3 = 2815 Hz), differing F0s resulted in slight variations in formants (on the order of 30 mels). Low-pass filtered white noise (0–2.5 kHz for simple tones, 0–10 kHz for harmonic stacks, 0–5 kHz for vowels) was presented at a level of approximately 60 dB SPL (participants were permitted to adjust noise volume) continuously throughout threshold and target-probe procedures. Participants listened through Sennheiser HD595 headphones while seated in an ETS-Lindgren soundproof chamber and stimulus delivery and response collection was controlled from a Windows computer (ASUS Xonar Essence STX sound card) with custom software written in MATLAB.

### Participants

**Fig 2:**
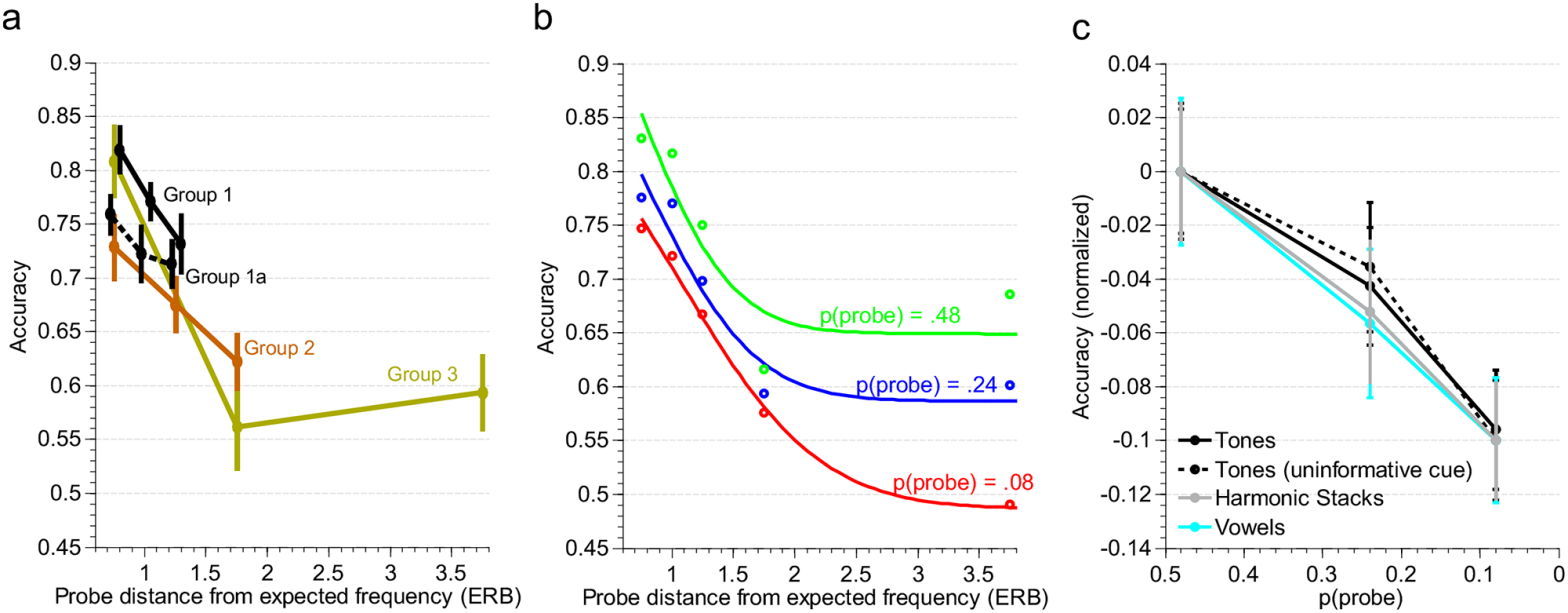
Attention-driven and prior-driven adaptation reflected in tone detection accuracies. (a) Tone attentional band effects for four separate subject groups. The different groups are slightly offset horizontally for ease of visualization of error bars. (b) Independent effects of probe frequency distance from the cued frequency and prior probability of the probe frequency aggregated across groups 1, 2, and 3. Circles represent average listeners’ data, solid lines represent a fit with a sum of two Gaussians. (c) Prior probability alters sensitivity similarly across stimulus classes and for informatively and uninformatively cued tones. “Accuracy for each study was normalized to the accuracy associated with the most accurate condition (i.e. highest probe probability) within that study such that all plots begin at 0.”

Seventy-seven naïve volunteers (age range = 18–33; mean age = 21; 51 females) self-identified as having no hearing difficulties successfully completed testing (six additional volunteers were not included due to incomplete data sets). The experiments were conducted in accordance with the declaration of Helsinki and after approval by the Johns Hopkins University Homewood institutional review board. All subjects gave written informed consent to participate in the study and were compensated $20 for participation in threshold and probe-signal procedures lasting two hours. Volunteers were assigned to one of several identical probe-signal tasks that used matched stimuli of different acoustic classes as summarized in Table 1: sinusoidal tones with informative cues (N = 15, Groups 1) sinusoidal tones with uninformative cues (N = 15, Group 1a), harmonic stacks (N = 15, Group 4), and vowels (N = 10, Group 5). Collecting responses for a larger range of probe frequencies would have caused the experiment to run excessively long. To confirm that any results were consistent across a larger range of probe frequencies for which the attentional band is often estimated, volunteers for the basic sinusoidal informative cue version of the task were randomly assigned to one of three probe frequency cohorts: .75/1/1.25 ERB (N = 15, Group 1 described above), .75/1.25/1.75 ERB (N = 12, Group 2), and .75/1.75/3.75 ERB (N = 10, Group 3). Data collected across these three cohorts were analyzed both as separate experiments (as shown in Fig 2a and 2c), and as a collapsed dataset (as shown in Fig 2b), with similar results.

**Table 1:** Summary of different experimental conditions.

Group / Experiment	Stimulus Type	Attentional Cue Condition	Cue Fq (Hz)	Probe Fqs (Hz)			N
				Probe 1	Probe 2	Probe 3	
1	tone	Informative	250	291	305	319	15
1a	tone	Uninformative	777	291	305	319	15
2	tone	Informative	250	291	319	350	12
3	tone	Informative	250	291	350	487	10
4	harmonic stack	Informative	250	291	305	319	10
5	vowel (pitch)	Informative	250	291	305	319	15

### Model

The model replicated the two-interval forced choice detection paradigm by presenting two stimuli *s*_*1*_ and *s*_*2*_ to an array of *N* tonotopically-organized neurons. Both *s*_*1*_ and *s*_*2*_ were *N*×1 vectors representing *N* possible frequency channels whose activity is randomly drawn from a standard uniform distribution. One of the two stimuli had an additional activation at channel *k* representing a tone embedded in noise background. Here, *N* was arbitrarily set to 100 and *k* set *to* 30 for target frequency and 40,55,70,80,95 for probe frequencies. The tone amplitude was set at signal to noise ratio -11.5dB. Both stimuli were processed through *N* neurons; with Gaussian *f*_*i*_(*s*) = *N*(*i*,σ = 2) tuning curves (*i* = 1, …, *N*). Variations of the number of channels and shapes of tuning curves (within reason) did not quantitatively change the function of the model.

For each stimulus *s*_*j*_, neuron *i* fired *r*_*i*_ spikes following a Poisson process with mean *f*_*i*_(*s*). An estimated neural log likelihood function was obtained as *r*_*i*_*f*_*i*_(*s*); then integrated across all *N* neurons to yield an estimate of the stimulus log likelihood $\text{log}L_{r}\left(s\right)\approx \Sigma_{i=1}^{100}r_{i}f_{i}\left(s\right)$ following the implementations in [27,28]. The almost equality indicates that normalization terms, independent of the stimulus structure, are safely ignored from the computation. This likelihood function was then integrated with a prior distribution *π*(*s*) by summing the log of both quantities. We also explored alternative implementations for integrating priors with neural responses; by multiplying the tuning curve of each neuron with a scaled version of the prior distribution *π*(*s*). This case would mimic changes to the tuning properties of the neural array as a result of contextual prior accumulation. Computationally, both implementations were equivalent and yielded comparable estimates of the posterior probability Θ(*s*|*r*).

For the uninformative cue condition, the interval {*s*_1_,*s*_2_} with highest mode (highest maximum a posteriori estimator) was chosen as the interval with correct target or probe tone. For the informative cue case, a weighted posterior mean estimator with a bell shaped weight function *W*(*s*) was centered on the location of the cue, and modeled as *N*(*cue*,σ = 5). Again, the interval with the highest peak was flagged. For both informative and uninformative conditions, the location of the detected tone was used to update the distribution of priors (initially normalized to a uniform distribution). The update function used a temporal decay term (*α* = 0.12) such that ${\hat{\pi }}_{t}=\left(1-\alpha \right)\pi_{t-1}+\alpha \pi_{t}$. This parameter was a crucial component of the model and its value (in the range [0.075−0.15]) was found empirically to give the best qualitative match to behavioral data. No aspect of the model was fit to the behavioral data.

### Data Analysis

All statistics and analyses were calculated using Matlab (Mathworks, Inc.). Probe detection accuracies from simulated and behavioral data were analyzed with two-way repeated measures ANOVAs. Effect sizes are reported as partial eta squared (η^2^_p_) values. All error bars displayed are 1 standard error of the mean calculated for repeated measures [29]. Because of the inherent noise in the model (nondeterministic nature of background noise, probabilistic behavior of neurons), 100 Monte Carlo simulations were run, and the average across all runs were reported.

## Results

Effects of attentional and contextual prior probabilities on perceptual sensitivity. Replicating established tone-frequency attentional band effects, detection accuracies significantly declined with increasing frequency separation from the cued focus of attention [Main effect of probe distance from expected frequency: F_2,70_ = 18.03, P < .0001; Individual cohorts, Group 1, F_2,28_ = 5.72, P < 0.01; Group 2, F_2, 22_ = 4.74, P < 0.05; Group 3, F_2_,_16_ = 14.31, P < 0.001] (Fig 2a and 2b). Detection accuracies also significantly declined with decreasing probe probability [Main effect of probe probability: F_2,70_ = 25.46, P < 0.0001; Individual cohorts, Group 1, F_2,28_ = 13.15, P < 0.001; Group 2, F_2, 22_ = 5.55, P < 0.05; Group 3, F_2_,_16_ = 9.22, P < 0.01] (Fig 2b), indicating that short-term statistics associated with tone frequency were tracked by listeners, and sensitivity to different tone frequencies adapted according to prior probability. Interestingly, there was no significant interaction between probe distance and probe probability [F_4,56_ = 0.56, P = 0.69; Individual cohorts, Group 1, F_4,56_ = 0.03, P = .99; Group 2, F_4, 44_ = 0.52, P = 0.72; Group 3, F_4_,_32_ = 1.68, P = 0.18], suggesting that tone sensitivities were modulated by the collection of tone frequency priors *independently* from selective attention to the cued frequency.

Prior-driven adaptation in the absence of cued attention. If attentional and contextual prior probabilities are genuinely independent factors, the effects of probe probability reported above should be closely replicated even in the absence of an attentional manipulation. To test this claim, the procedure was replicated with an uninformative cue (at 777 Hz). The uninformative cue replaced the informative cue in all trials, and instructions made no mention of using the cue to guide attention, effectively eliminating any attentional prior (for this and all following results, only three of the five probe frequencies were tested). In the absence of cued attention, there was a clear effect of contextual priors on detection accuracy [main effect of probe probability, F_2,28_ = 13.18, P < 0.001]. Reductions in detection accuracy from the highest to the lowest prior probability condition were virtually identical in the informative and uninformative cue paradigms (Fig 2c), as were the associated effect sizes [informative cue: η^2^_p_ = .48; uninformative cue: η^2^_p_ = .48]. When informative and uninformative cue groups (Groups 1 and 1a) were directly compared, there was no group by probe prior interaction, [3-way ANOVA, repeated measures on two factors: main effect of probe probability, F_2,56_ = 0.851, P = 0.92], further suggesting that contextual priors condition sensitivity *independently* from attentional priors.

An additional finding of the uninformative cue manipulation was a non-significant trend for the effect of “probe distance” [main effect of probe distance, F_2,28_ = 2.69, P < 0.086] (Fig 2a, Group 1a). In previous experiments where attention was cued, “probe distance” referred to the frequency distance from the cued attentional target. However, there was no attentional target in the uninformative cue paradigm. Here, “probe distance,” can only refer to distance from the 250 Hz tone: the tone frequency with the highest contextual prior. In this context, an effect of “probe distance,” can be understood as a weak context-driven band, rather than the standard attentional band: a point we will further address in the discussion section. While it is possible that listeners noticed the high probability of the 250 Hz tone and explicitly attended to it, participants reported no awareness of this or any related strategy when interviewed.

Perceptual adaptation across stimulus classes. To test whether attentional and contextual priors associated with more complex acoustics could be tracked and used, the same informative cue procedure was repeated with harmonic spectra (Group 5) and vowels (Group 6), with target and probe pitches for these stimuli matched to the tone frequencies reported above. Significant main effects of probe prior were found for both classes of stimuli [harmonic: F_2,28_ = 14.96, P < 0.0001; vowels: F_2,18_ = 11.52, P < 0.001], and the sizes of these effects were similar to those reported above [harmonic: η^2^_p_ = .52; vowels: η^2^_p_ = .56] (Fig 2c). No attentional bands were found for these stimuli [harmonic: F_2,28_ = 1.85, P = 0.18; vowels: F_2,18_ = 0.98, P = 0.91], indicating that attentional and contextual priors behave differently and suggesting different mechanisms for their effects on perception.

Listeners collect priors near-optimally. Prior probabilities can be time-invariant, such that new information is integrated with the entire unweighted historical record. Alternatively, they can be time-dependent, such that prior distributions are either estimated for a discrete and limited historical window, or weighted to place more importance on recent experiences. A weighting of the past would be optimal if it gives the most predictive estimate of the future. Here, an optimal integration window size was defined as the number of preceding trials (*w*) that yielded, on average, the most accurate (best correlated) estimates of the underlying probe priors used to generate trials in the informative cue tone paradigm. Using the actual trial sequences participants experienced, probe prior estimates were made for each trial, for each participant, and for each *w* between 5 and 150 preceding trials.

**Fig 3:**
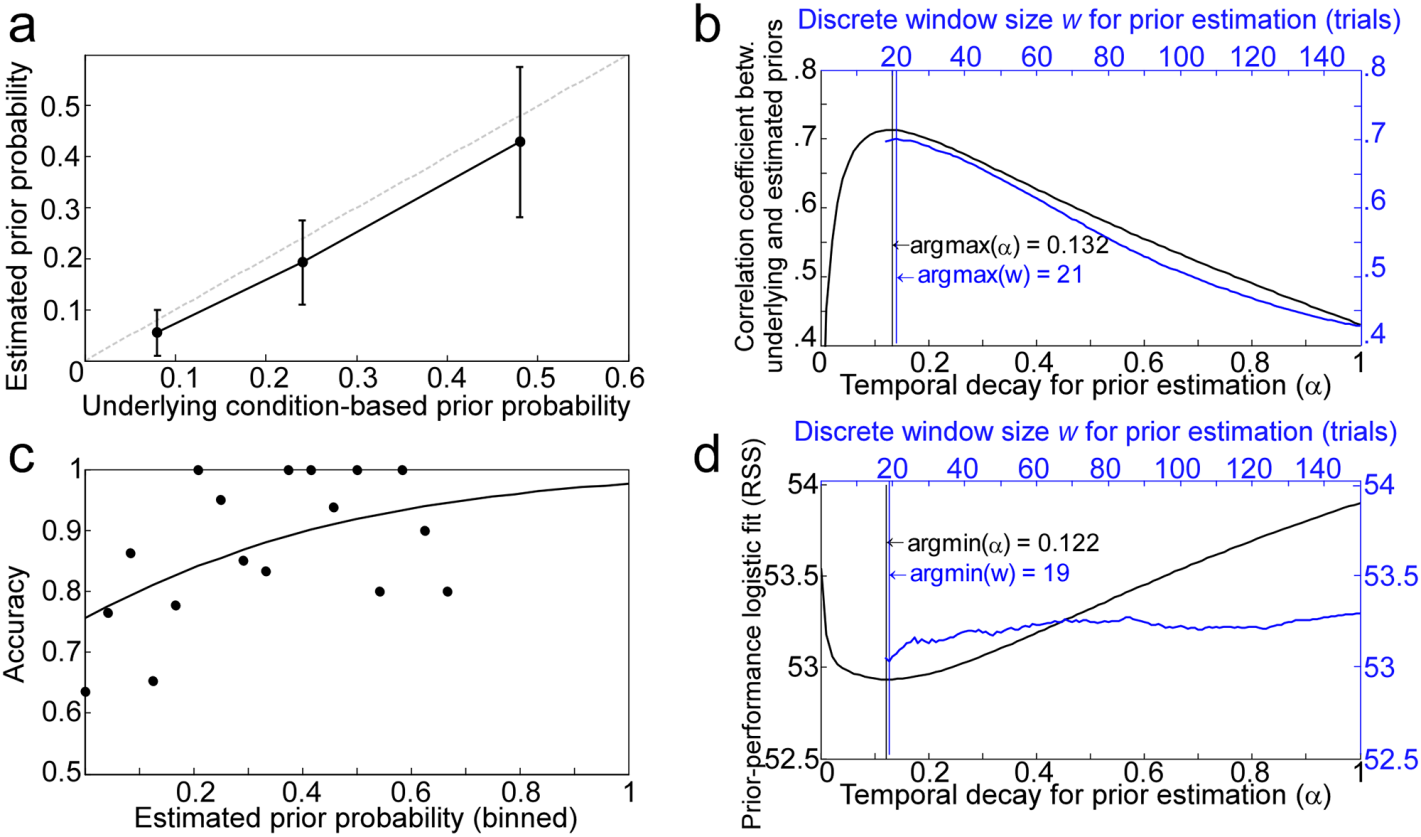
Temporal properties of prior estimation. (a) Relationship between the average underlying probe priors and average estimated priors for one subject using an window of ***w*** = 21 trials. (b) Results of aggregating the correlation shown in (a) across different subjects and different window sizes (or α values). The argmax values refer to the window sizes and α values that resulted in maximum correlation coefficients for the probe priors estimated from correct trials. (c) Data (circles) and logistic regression fit (solid line) between detection accuracies and prior probabilities estimated for one subject using a window size of ***w*** = 19 trials. Priors were put in discrete bins for illustration, but were treated as a continuous variable in all analyses. (d) Results of aggregating the fits (RSS, residual sums of squares) like the one shown in (c) across different subjects and different window sizes (or α values). The argmin values refer to the window sizes and α values that resulted in the minimum fit errors for the probe priors estimated from correct trials.

Correlations between estimated and actual priors (Fig 3a) were then averaged across participants for each window size *w* (Fig 3b). Calculating probe priors using the preceding 21 trials produced the best average estimates of underlying probe priors. Note that this number is specific to this paradigm and experimental design.

We also estimated the most likely window size *actually* used by listeners. Given the main result of a positive relationship between performance and prior, the estimated probe priors for the different window sizes were used to fit logistic models relating probe prior with probe detection accuracy: one model for each participant for each window size (Fig 3c). Window sizes that better predicted performance, based on residual sums of squares, were considered closer to the actual or *functional* window sizes used by listeners. The window size resulting in the best fit, averaged over subjects, was deemed the average window size most likely used by listeners to estimate priors. This analysis suggests that participants used an average window size of 19 preceding trials in estimating probe statistics, a figure close to the optimal window size reported above (Fig 3d).

Different assumptions about how priors are collected will yield different estimates of optimal and actual window sizes. For example, it is unclear whether a model should assume that priors get updated on when the listener does not accurately hear the tone. Because the task involved a two-interval forced choice, it is difficult to recover whether the listener accurately heard the tone on a trial-by-trial basis: incorrect trials indicate that the listener may not have accurately heard the tone, and correct trials are made up of both detections and guesses. To test different assumptions about when priors might be updated, we calculated actual and optimal window sizes for models where priors were updated on all trials, on correct trials, and on estimated or rectified correct trials that took into account the fact that some correct trials were guesses. We used individual accuracy rates for each subject (for each level of probe distance and probe prior), to rectify for correctly scored guesses. In this way, prior estimates were updated only on a corrected proportion of correctly scored detections. In addition, since probe priors were inversely proportional to target priors (i.e. blocks with more probes had fewer targets), identical analyses were performed using targets to calculate prior probability. The model that computed priors based on only the truly detected probe trials (i.e. rectified correct probes) had the best overall agreement with the window size actually used by listeners (as shown in Table 2).

**Table 2:** Values that resulted the optimal (maximum correlation coefficients between underlying and estimated priors) and functional (minimal prior-performance logistic fit error) window sizes and decay rates. Optimal and function parameters are similar, regardless of prior estimation method.

	Window size (trials)		Decay rate (α)	
	Optimal *w*	Functional *w*	Optimal α	Functional α
All probes	19	12	0.08	0.08
Correct probes	22	12	0.1	0.13
Rectified correct probes	21	19	0.13	0.12
Correct targets	29	12	0.07	0.12
Rectified correct targets	25	19	0.09	0.15

In lieu of discrete windows, the distribution of priors can also continuously decay over time such that the more recent information is, the more it contributes to the distribution. The same analyses reported above were repeated using a temporal decay coefficient (α) to estimate probe priors and determine the optimal and functional decay weights. The coefficient α refers to the weight of the most recent trial relative to the historical prior distribution, such that smaller values closer to 0 correspond to slower decay rates (long memory) and higher values closer to 1 correspond to higher decay rates (short memory). This coefficient is used in computing prior probability, *π*(*t*,*f*), where *t* is a time point or trial, and *f* is a tone frequency: $\hat{\pi }\left(t,f\right)=\left(1-\alpha \right)\pi \left(t-1,f\right)+\alpha \pi \left(t,f\right)$, where Σ_*f*_*π*(*t*,*f*) = 1. Similar to the integration window results reported above, the optimal and functional α values were very similar: 0.132 and 0.122, respectively (Fig 3c and 3d).

Because the discrete window-based and continuous decay-based models yielded almost identical best fits, these results cannot endorse one of these representations of priors over the other; though both models agree that the average listeners appeared to be collecting a distribution of priors optimally over the course of this experiment. Furthermore, optimal window sizes and temporal decay coefficients were not manipulated, and so whether the functional window sizes or decay rates used by participants would track different optimums, and how robust these results are to different presentation paradigms, cannot be determined from this data.

**Fig 4:**
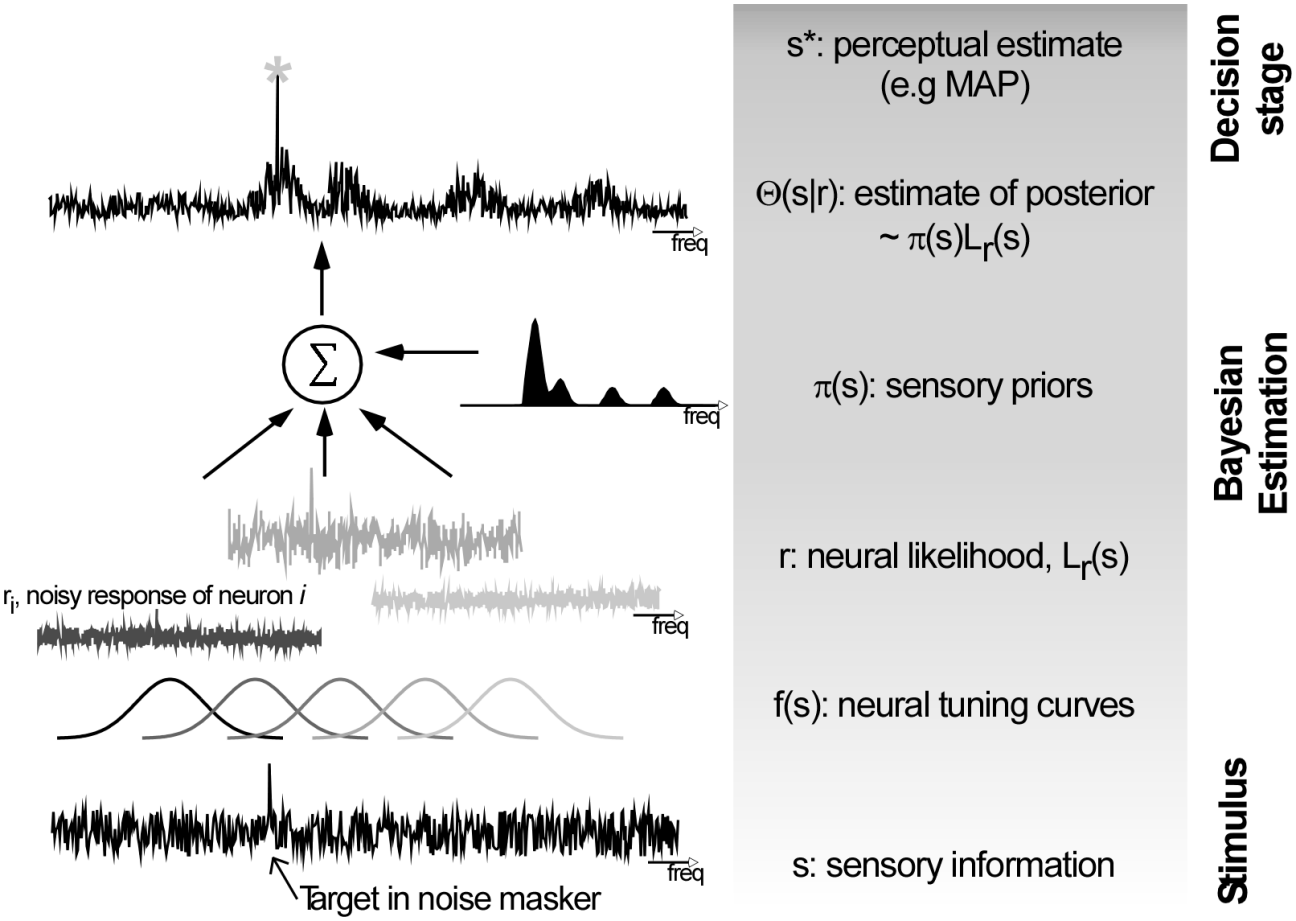
A Bayesian model of tone detection in noise. The sensory input (bottom) is processed through an array of nondeterministic neurons, and yields a neural likelihood function ***L***_***r***_(*s*). This likelihood is integrated with an estimate of contextual priors ***π***(***s***) to yield an adjusted estimate of the sensory posterior. A Bayesian decision determines the likely location of the target.

A Bayesian Observer model accounts for attentional and perceptual adaptation. The model tests the intuition that the behavioral results reported here can be captured by an optimal decision rule based on a Bayesian regime. The model estimates the detectability of a tone embedded in a noise background (Fig 4). Given sensory input *s*, an array of *N* tonotopically-organized non-deterministic neurons produce r_i_,{*i* = 1, …, *N*} spikes over the duration of the stimulus. The firing rate of each neuron *i* follows Poisson statistics and reflects the match between the input and the neuron’s tuning curve *f*_*i*_(*s*). The ability of the perceptual system to detect a target in the stimulus depends on the fidelity of its sensory mapping; i.e. its ability to give the best estimate of the stimulus from a readout of an inherently noisy neural population. An estimate of the likelihood of stimulus *s* based on the population response of *N* neurons is modeled as a weighted integration of responses of individual neurons into a single observation, or likelihood function *L*_*r*_(*s*). This function, or its equivalent log likelihood, was previously shown to yield an optimal decoding of sensory information given noisy measurements [27,28,30]; and is obtained by adding the responses of each neuron *r*_*i*_ weighted by its corresponding tuning function *f*_*i*_(*s*).

In a frequentist non-Bayesian setting, this function gives an estimate of likely stimuli that could have given rise to the observed population response. Its peak (i.e. location with highest likelihood) corresponds to the expected position of the target to be detected (i.e., a maximum likelihood estimate) [31,32]. It amounts to a homogeneous detection of both target and probe frequencies; assuming equi-probable priors, uniform activity and tuning properties across the tonotopic axis. In contrast, a Bayesian setting combines this sensory likelihood function with prior beliefs about the stimulus in order to guide the perceptual decision. In the current model, the likelihood of the stimulus is integrated with prior beliefs about the stimulus *π*(*s*) to yield a posterior estimate of the probability of *s* given the observed neural response:Θ(*s*|r) ∝ *L*_*r*_(*s*)*π*(*s*).

Once a posterior is estimated, a perceptual decision uses a selection rule to determine the presence of a target note embedded in the noise. In case of complete uncertainty (i.e. uninformative cue), the decision stage relies on a uniform decision function. Here, we use a standard 0–1 loss function (i.e. ${\text{lim}}_{\varepsilon \to 0}arg\;max_{s\prime }\overset{s^{\prime }+\varepsilon }{\underset{s^{\prime }-\varepsilon }{\int }}\Theta (s|r)ds$), which corresponds to a maximum a posteriori (MAP) estimator [32]. Given the two-interval forced choice structure of our paradigm, the interval with the highest MAP value is designated as the one likely to contain the tone embedded in noise. Conversely, in presence of limited uncertainty with an informative cue, the decision model integrates the information about the cued frequency of a tone embedded in noise by using a weighted posterior mean estimator. Additional model details can be found in Methods.

**Fig 5:**
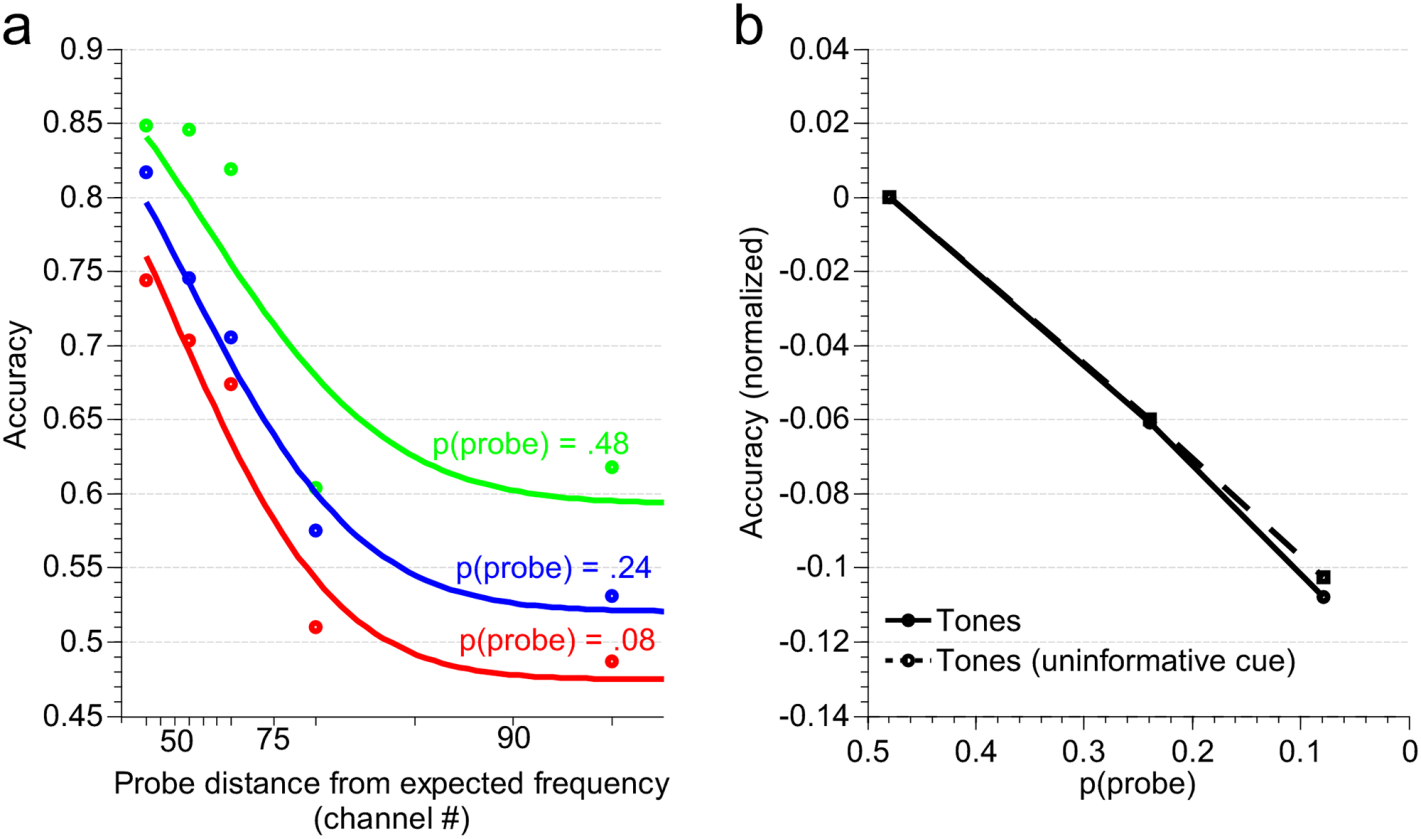
Bayesian model predictions of detection accuracies. (a) Effects of probe distance and probe prior (informative cue). Circles represent average simulation data, solid lines represent a fit with a sum of two Gaussians. (b) Model shows normalized accuracies for informative and uninformative cue conditions for tone detection.

The model behaved similarly to the human listeners (Fig 5a), yielding a significant main effect of probe distance relative to target [ANOVA: F_4,396_ = 1372, P < .0001] as well as main effect of probe probability [F_2,198_ = 902.6, P < .0001], with no significant interaction [F_8,792_ = 0.8, P = 0.2] suggesting that integration of probe priors is independent of probe distance effects. In addition, the model replicated prior-driven adaptation in absence of cued attention; and yielded similar contextual prior effects with and without informative cue (Fig 5b). The model also supports that experimental observation that listeners are collecting priors over a near optimal analysis window or past history. Both model and behavioral data *independently* suggested that integrating priors in a time-dependent fashion with a decay parameter in the range of 0.1–0.15 yielded the best interpretation of the tone accuracy performance with time-varying probe probabilities.

## Discussion

We investigated the role of explicit attention-driven and implicit context-driven expectations of frequency in the detection of tones and vowels. As expected, placing a high attentional prior on one particular tone frequency reduced sensitivity to other probe frequencies proportional to their frequency distance from the cue. Remarkably, sensitivity was influenced just as strongly by the implicit prior probabilities of the tone frequencies, and this effect extended to pitches of complex tones and vowels.

While we observed both attention-driven and context-driven adaptation, perceptual sensitivity adapted to contextual priors independently of any attentional prior. There was no interaction between these factors in any of the experimental or modeling results: contextual priors had the same effect on detection sensitivity whether there was an effective attentional prior, an ineffective attentional prior, or no attentional prior at all. Computationally, attentional and contextual priors can be treated interchangeably [33], but results reported here suggest that attention-driven and context-driven adaptations are complementary processes (that may or may not target the same representations). This result has implications for the treatment of contextual priors in both the neural and psychometric literatures.

A proposed model, operating in Bayesian regime is able to qualitatively replicate behavioral responses, hence substantiating suitability of the Bayesian interpretation of the role of priors in modeling attentional sensitivity and shaping perceptual decisions. The Bayesian framework permits an integrated assessment of two key components of the task at hand: the integration of sensory evidence with learned statistical priors, as well as a higher-level strategy for target detection in noise. An alternative account based on signal detection theory [24,31] could also be considered here, but such detection-based models inherently dissociate the representation of evidence from the decision rules. This dissociation limits our ability to gauge time scales of integration of priors, as well as the potential contribution of recent probe history.

### Neural representation of contextual priors

According to studies of attention-driven neural adaptation, attention shapes perception by transiently altering the response properties of sensory [34] or object representations [35] in a “top-down” fashion [36]. In auditory detection tasks similar to the one used here, auditory cortical receptive fields rapidly adapt to favor detection of attended tone frequencies through sharpening and gain control [37–40]. Context-driven adaptation might involve the same mechanisms. While it is not yet known where or how contextual priors are neurally represented or integrated with incoming sensory information, contextual priors could be reflected in the sharpening and gain control of auditory receptive fields, just as attentional priors are thought to be. Though they mirror sensory history, these priors are not necessarily purely “bottom-up”: they could reflect either early pre-detection “bottom-up” sensory likelihoods known to induce habituation or sensitivity shifts in sensory responses [41], or alternatively, they could reflect “top-down” post-detection estimates. Our experimental paradigm cannot disentangle the relative involvement of these two possibilities. It is worth noting that the nature of the paradigm operating near threshold with ongoing noise structure likely obscures any effects of sensory habituation. The model presented here implemented a post-detection estimate of priors, effectively separating contextual priors from neuronal tuning curves. Alternative schemes, like the encoding of priors in the size of sensory representations [42,43], have been proposed for encoding more stable, long-term contextual prior distributions.

### Psychometric treatment of contextual priors

Psychometric studies characterize selective frequency listening with an auditory filter function in which the effective attenuation (in dB) of a probe tone is proportional to its frequency distance from the expected or target tone [20]. Additional factors are thought to determine the width and gradient of the filter, such as the level of masking noise, and the frequency range of probe tones [44]. It is tempting to simply add probe priors to the list of factors that modulate the shape of the attentional band, but that may well be the tail wagging the dog.

Alternatively, the attentional band in auditory frequency listening could be characterized as a special case of modality-general *perceptual* bands. Perceptual bands—perceptual attenuation proportional to the distance from relatively expected feature levels—can be understood as a simple consequence of the kind of attentional *or* contextual prior integration along a feature axis (e.g. tone frequency, direction of visual motion) described in this report and others [8,45,46], as the shape of the perceptual band will directly reflect the shape of the prior probability distribution.

To illustrate this concept, consider a simple attentional prior represented as some bell-shaped distribution along the frequency axis. According to the distribution, prior probability falls off proportional to the distance from the most likely frequency. Integrating this distribution with incoming sensory information results in a bell-shaped perceptual band of tone frequency sensitivities. Certainly prior distributions will often be more complex than this simple attentional prior, but perceptual bands can form at any local maximum in the prior distribution through a variety of mechanisms. For example, high priors associated with particular levels of a feature can act as perceptual magnets or attractors in feature estimation [13], producing bands in the prior distribution, and in turn, perceptual bands in sensitivity. Other aspects of sensory processing, such as bell-shaped noise distributions, or filters in the sensory processing stream [47], can also lead to bands in prior distributions. One caveat to this understanding of perceptual bands is that the neural representation of sensory features may take more complex forms than their physical instantiation, which distorts the shape of prior-driven effects. For instance, our data reveals no attentional band effects for harmonic stack and vowel stimuli. The neural and perceptual spaces for both features are far more complex than one simply anchored by just tonotopy [48–51], potentially masking any perceptual bands along a frequency axis.

Others have interpreted perceptual bands as evidence for explicit attention, but as suggested here, perceptual bands may occur without observer awareness. In their study of priors associated with direction of motion, Chalk and colleagues [8] reported that viewer judgments were biased by implicitly learned directional expectations, and faster and more accurate motion detections occurred when stimuli moved in expected directions. Similar to effects reported here, they found perceptual bands of sensitivity around the most expected directions of motion, but proposed that, “learned expectations led participants to direct selective attention towards the expected stimuli.” While we cannot entirely rule out that our participants directed selective attention to tone frequencies in accordance with their prior probabilities—only that they were unaware of doing so at the time of their post-test interview—others have shown strong evidence for subliminal sensitivity to stimulus history [52]. Regardless, our findings indicate that these contextual priors (whether explicit or implicit) operate as independent mechanisms from those of explicit attentional priors. Given that equivalent perceptual bands can result from integrating priors observers are aware of *or* priors they are naïve to, it may not be necessary to appeal to selective attention (in the traditional sense of a conscious, explicit focus of attention) to explain these results. What’s more, it may be necessary to control for the effects of implicit contextual priors when interpreting studies of selective attention.

The effect of priors on perceptual sensitivity likely extends beyond relatively simple perceptual bands. Although we used prior probability distributions limited to a single perceptual dimension, studies of selective attention suggest that auditory perception is sensitive to multimodal and joint prior distributions. Perceptual bands have been observed when priors are spread across four different tone frequencies [23], suggesting that more complex prior distributions can be represented and used to modulate sensitivity. In addition to tone frequency, expectations about tone duration can modulate sensitivity, facilitating perception of tones that have an expected duration relative to tones with unexpected durations. Importantly, when expectations about tone frequencies and tone durations are manipulated jointly (e.g. cueing a long, low frequency tone), listener sensitivities reflect the joint prior distribution [53].

### Perspectives

While there are many indications that complex, multidimensional prior distributions can be represented and used to improve perception, several issues must be addressed before this work can be extended to perception of real-world or natural scenes. First, the vast majority of related studies have measured prior-based perceptual adaptation only at near-threshold levels. As a result, the degree to which priors influence the speed and accuracy of perception at more typical levels is unknown. Second, to fully exploit the statistical structure in the environment, encoded prior distributions should incorporate contextual information (e.g. expectations about the next token in a potential series). Context-specific statistical learning has been widely demonstrated [54,55], but whether it is a factor in sensory perception is unknown. Third, listeners in the current study learned to use close-to-optimal historical window sizes or decay rates in representing priors, but it is unclear whether listeners are optimally sensitivity to the rates of prior fluctuation in real environments. Finally, sensitivity was relatively increased for more likely feature levels and decreased for less likely feature levels in the kind of perceptual adaptation discussed here, but attentional capture of rare, unexpected, or oddball events has also been widely observed [56]. Furthermore, tones presented further from expected frequencies can induce higher degrees of attention capture [57], somewhat counter to results presented here. It will be important to resolve how perception of expected sensory information is facilitated relative to unexpected information, while still allowing for attentional capture during some expectation collisions.

## Acknowledgments

This research was supported by National Science Foundation grant IIS-0846112, National Institutes of Health grants 1R01AG036424 and T32 DC 23–27, and Office of Naval Research grants N00014-12-1-0740 and N000141010278. The funders had no role in study design, data collection and analysis, decision to publish, or preparation of the manuscript. The authors thank research assistants Katherine Simeon and Nicha Apichitsopa for their valuable suggestions and contributions to data collection.
